# Induction of Inflammation In Vivo by Electrocardiogram Sensor Operation Using Wireless Power Transmission

**DOI:** 10.3390/s17122905

**Published:** 2017-12-14

**Authors:** Jin-Chul Heo, Beomjoon Kim, Yoon-Nyun Kim, Dae-Kwang Kim, Jong-Ha Lee

**Affiliations:** 1 Department of Biomedical Engineering, School of Medicine, Keimyung University, Daegu 42601, Korea; washingbuffer@gmail.com; 2 Department of Electronic and Electrical Engineering, School of Engineering, Keimyung University, Daegu 42601, Korea; bkim@gw.kmu.ac.kr; 3 Department of Internal Medicine, Dongsan Medical Center, Keimyung University, Daegu 41931, Korea; ynkim@dsmc.or.kr; 4 Department of Medical Genetics, Hanvit Institution for Medical Genetics, Keimyung University, Daegu 42601, Korea; dkkimmd@kmu.ac.kr

**Keywords:** biocompatibility, electrocardiogram, implantable sensor, wireless power

## Abstract

Prolonged monitoring by cardiac electrocardiogram (ECG) sensors is useful for patients with emergency heart conditions. However, implant monitoring systems are limited by lack of tissue biocompatibility. Here, we developed an implantable ECG sensor for real-time monitoring of ventricular fibrillation and evaluated its biocompatibility using an animal model. The implantable sensor comprised transplant sensors with two electrodes, a wireless power transmission system, and a monitoring system. The sensor was inserted into the subcutaneous tissue of the abdominal area and operated for 1 h/day for 5 days using a wireless power system. Importantly, the sensor was encapsulated by subcutaneous tissue and induced angiogenesis, inflammation, and phagocytosis. In addition, we observed that the levels of inflammation-related markers increased with wireless-powered transmission via the ECG sensor; in particular, levels of the Th-1 cytokine interleukin-12 were significantly increased. The results showed that induced tissue damage was associated with the use of wireless-powered sensors. We also investigated research strategies for the prevention of adverse effects caused by lack of tissue biocompatibility of a wireless-powered ECG monitoring system and provided information on the clinical applications of inflammatory reactions in implant treatment using the wireless-powered transmission system.

## 1. Introduction

Implantable wearable devices have various applications and advantages and are convenient for the user, allowing acquisition of continuous data compared to portable units. In particular, wearable equipment is useful for identifying and controlling the continuous care of patients with critical conditions, such as diabetes or heart diseases [1,2]. However, several problems are associated with the transplantation of such devices, such as inflammation, rejection, and apoptosis within the body [3,4]. The rejection of foreign material causes inflammation and phagocytosis by responding immune cells [5]. The foreign body reaction is the final stage of inflammation, and several studies have attempted to overcome this issue while designing biomaterials or medical devices [6,7]. Maintaining operating conditions and ensuring material biocompatibility is crucial for using implantable electronic devices. However, currently, tissue biocompatibility of electronic devices in vivo is insufficient.

Recently, implantable wearable devices with external power supply have been developed [8] to overcome the risk of inserting a battery-powered device [9]. Although battery technology has significantly advanced in terms of stabilization, concerns regarding the insertion of batteries in humans persist. To overcome this problem, implementation of wireless charging technology for wearable devices has been attempted [10]. However, the safety of using a wireless power supply for machine operation in a living body remains unknown.

Wireless-powered transmission devices consist of equipment that supply power to the device, and various tissues are exposed to the effects of the electric field generated when the device receives power [11]. Continuous exposure to an electrical field elicits various side effects [12]. However, studies on the efficiency and safety of implantable wireless-powered transmission apparatuses for the development of various implantable diagnostic devices are rare. In addition, in vivo studies addressing the safety of operational wearable devices are lacking. Although several studies have investigated the tissue compatibility of implants consisting of different types of sensor material, few studies have examined the operation of the sensor.

Therefore, the purpose of this study was to determine the biocompatibility of an electrocardiogram (ECG) sensor under operating conditions using an in vivo rat model. We assessed the surrounding tissue of the inserted sensor and evaluated the level of inflammation and cellular damage by using histology, transmission electron microscopy (TEM), and mRNA analysis. Our findings indicated that the sensor powered by a wireless power source induced an inflammatory response and increased the number of apoptotic cells in the surrounding tissue compared to the nonoperational sensor.

## 2. Materials and Methods

### 2.1. Insertion and Measurement of the ECG Sensor

First, we describe the design of the wireless-powered sensor [13]. A micro-sized implantable ECG sensor was developed to provide high diagnostic yield. The dimensions of the integrated sensor were approximately 0.30 cm × 3.0 cm × 0.48 cm. The proposed ECG sensor had voltage and power of 7.3 V and 26.6 mW, and noise generation was inversely proportional to the length of the wireless communication antenna inside the sensor. The radio and power transmission frequency was 13.56 MHz.

**Figure 1:**
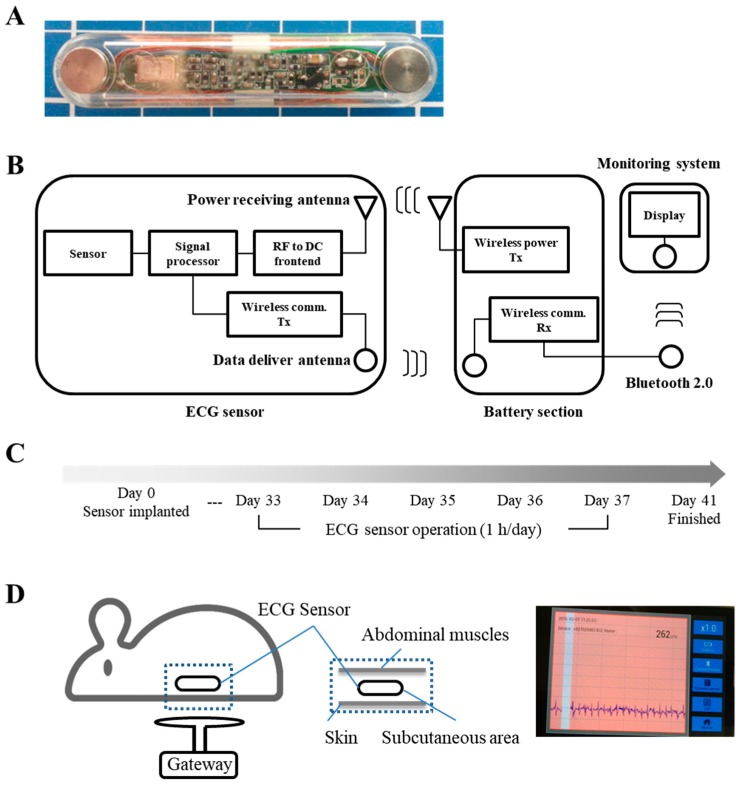
(**A**) An ECG sensor with the electrode of the anode; (**B**) Schematic view of the system. Dual antenna structure of a wirelessly powered sensor; (**C**) Schematic representation of the model for experimental conditions; (**D**) An ECG sensor was implanted subcutaneously within the rat, and the ECG signal was checked using a wireless charging and Bluetooth system.

In total, 15 healthy adult male Sprague Dawley rats (Hyochang Science, Daegu, Korea) weighing 380–460 g were randomly divided into three groups of five rats each, namely, the implantation control, sensor operation, and sensor inactive treatments. The rats were housed in individual cages and maintained under a 12-h light/dark cycle with food and water available *ad libitum*. All efforts were made to minimize the number of animals used and limit animal suffering. The animals were anesthetized with 5% isoflurane (JW Pharm, Seoul, Korea), which was maintained throughout the surgical procedure. After inserting five sensors per group between the peritoneal epithelium and the skin, the surgical site was allowed to heal for a period of approximately 4 weeks. The study protocol was performed in accordance with the guidelines outlined in the Declaration of Helsinki and was approved by the Ethic Committee of Keimyung University (approval number: KM-2015-20R1). The wireless-powered ECG sensor was composed of three parts: (1) the ECG sensor implanted into the living body; (2) the battery section, which supplied power from the outside; and (3) operation of sensor and monitoring system using Bluetooth. The in vivo sensor received the power and delivered the acquired ECG signal from the electrodes at both ends. The wireless power system received signals from the sensor containing the ECG electric power supply to transmit to an external monitor via a Bluetooth system. The display system consisted of an ECG signal output on a smartphone (Figure 1A,B). After insertion of the sensor, the operation conditions were determined by checking the ECG and confirming the functioning of the wireless charging kit. Four weeks later, the sensor was operated for 1 h/day for 5 days between 33 and 37 days post-surgery. We checked whether the ECG signal was detectable from the rat (Figure 1C,D), and samples were collected on four days following the final sensor operation. The animals were sacrificed using CO_2_ gas, the status of the sensors was checked intraperitoneally, and tissues were collected from the area surrounding the sensors.

### 2.2. Histological Analysis

Histological examination was performed to determine infiltration of inflammatory cells. Samples for optical microscopy were fixed with 10% paraformaldehyde in 0.1 M phosphate-buffered saline (PBS; pH 7.4) and embedded in paraffin as previously described. Tissue sections were subjected to staining with hematoxylin and eosin (H & E) to observe general morphology [14]. Samples for TEM were fixed in 2.5% glutaraldehyde in 1× PBS, washed, incubated in 1% OsO_4_ for 1 h at room temperature, dehydrated with increasing concentrations of ethanol, and then embedded in Epon812 medium (Polysciences, Inc., Hirschberg an der Bergstraße, Germany). Ultrathin sections (70 nm) were subsequently collected on holy formvar-coated grids, contrasted with uranyl acetate and lead citrate, and finally examined using TEM (Hitachi, Tokyo, Japan). Total cell analysis was performed by comparing the working and not working group by H & E staining using paraffin blocks. Positive cell numbers were calculated as values per millimeter of subcutaneous tissue.

### 2.3. Reverse Transcription-Polymerase Chain Reaction (RT-PCR) Analysis of Gene Expression

The mRNA levels of inflammatory cytokines were analyzed by RT-PCR. Total RNA was extracted using TRIzol reagent (Molecular Research Center, Cambridge, UK) according to the manufacturer’s protocols. RNA (1–10 μg) from rat skin samples containing subcutaneous tissue was transcribed into first-strand cDNA with random primers in a reaction volume of 1 μL using an RT Master Mix (Takara, Nojihigashi, Japan), and 100 ng RT product was used as the PCR template. Quantitative PCR was performed with the cDNAs, SYBR Green I dye (SYBR Green PCR master mix; Takara, Nojihigashi, Japan), and primer sets for *Ifng* (sense: 5′-GATCCAGCACAAAGCTGTCA-3′, antisense: 5′-GACTCCTTTTCCGCTTCCTT-3′), *Il12* (sense: 5′-AGGTGCGTTCCTCGTAGAGA-3′, antisense: 5′-CCATTTGCTGCATGATGAAT-3′), *Il17* (sense: 5′-ATCAGGACGCGCAAACATG-3′, antisense: 5′-TGATCGCTGCTGCCTTCAC-3′), *T-bet* (sense: 5′- CCAACAATGTGACCCAGATGAT-3′, antisense: 5′-CTGGCTCACCGTCATTCA-3′), *IL-13* (sense: 5′-AGTCTTCAGTTTAAGCCAGCTTAC-3′, antisense: 5′-TTTTCAATGGAAGGTACCACAGCGG-3′), *Ifna* (sense: 5′-TCTGTAATGACCTCCAGCAGC-3′, antisense: 5′-CTGGGTCAGGGGAGATTCCT-3′), *Ifnb* (sense: 5′-ACTTGGGTGACATCCACGAC-3′, antisense: 5′-GGACCACCATCCAGGCATAG-3′), *TGF-β* (sense: 5′-TGGCGTTACCTTGGTAACC-3′, antisense: 5′-GGTGTTGAGCCCTTTCCAG-3′), *Tnfa* (sense: 5′- AAATGGGCTCCCTCTCATCAGTTC-3′, antisense: 5′- TCTGCTTGGTGGTTTGCTACGAC-3′), *Il4* (sense: 5′-CGTGATGTACCTCCGTGCTT-3′, antisense: 5′-GTGAGTTCAGACCGCTGACA-3′), *Il5* (sense: 5′-GGATGCTTCTGTGCTTGAACG-3′, antisense: 5′-CACTGTGCTCATGGGGATCT-3′), *Il6* (sense: 5′-TCCTACCCCAACTTCCAATGCTC-3′, antisense: 5′- TTGGATGGTCTTGGTCCTTAGCC-3′), *Il21* (sense: 5′-TCATCAACGACTTGTTGGCAC-3′, antisense: 5′-GCATTTAGCCATGTGCCTCT-3′), and *Gapdh* (sense: 5′-GTATTGGGCGCCTGGTCACC-3′, antisense: 5′-CGCTCCTGGAAGATGGTGATGG-3′). PCR was performed in a two-step reaction (95 °C for 30 s, 60 °C for 30 s) for 45 cycles after initial denaturation (95 °C, 15 min) using a Takara Thermal Cycler Dice Detector System (Takara). Relative expression of target genes was analyzed using the ΔΔCT method. Specific mRNA levels were quantified at points at which the system detected uptake in the exponential phase of PCR accumulation and were normalized to *Gapdh* mRNA levels [15].

### 2.4. Statistical Analysis

Data are represented as the mean ± standard deviation. Statistical significance was determined by Student’s t-tests and analysis of variance (ANOVA) in Microsoft Excel. Differences with *p* values < 0.05 and 0.01 were considered statistically significant.

## 3. Results

Here, we describe the design of the wireless-powered sensor. A micro-sized implantable ECG sensor was developed to provide high diagnostic yield (Figure 1A). A sensor node may take advantage of a radio frequency identification (RFID) system, which uses a double loop coil-shaped electromagnetic induction method-type wireless-powered transmission system. The ECG sensor had a voltage and power of 7.3 V and 26.6 mW, respectively, and a power transmission frequency or 13.56 M Hz (Figure 1B). The ECG sensor was inserted into the subcutaneous layer, after the surgical site was completely healed, and the sensor was operated using wireless-powered transmission for 1 h/day for 5 days, and ECG signaling checked (Figure 1C,D).

### 3.1. The Wireless ECG Sensor Affected the Biocompatibility of the Surrounding Tissue

**Figure 2:**
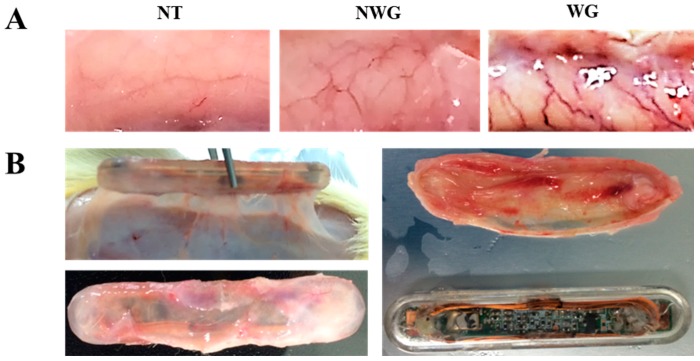

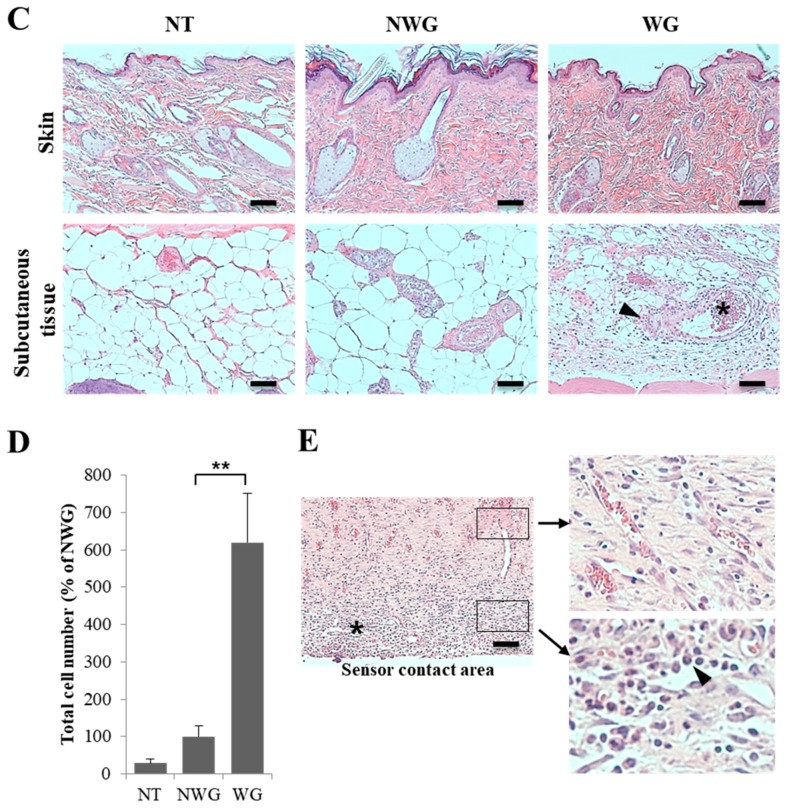
(**A**) Angiogenesis was induced in the skin by a functioning ECG sensor (NT, normal; NWG, not working group; WG, working group); (**B**) Sensor status of subcutaneous tissue according to wireless-powered transmission and sensor operation; (**C**) Hematoxylin and eosin staining of the skin and subcutaneous tissues showed that the sensor induced infiltration of inflammatory cells into the subcutaneous tissue. A number of inflammatory cells were observed in blood vessels (asterisk) and the surrounding tissues (arrowhead) (NT, normal; NWG, not working group; WG, working group); (**D**) Cell infiltration was increased by wireless-powered transmission; (**E**) There were numerous inflammatory cells in the contact area of the sensor (asterisk), and blood vessels were formed on the back side of sensor. Right insets show enlarged images of surrounding tissues (asterisk, inflammatory cells; arrowhead, eosinophil; scale bar, 100 μm). Significant difference from NWG, ** *p* < 0.01.

After insertion of the sensors, the biocompatibility of the implant was checked by histological analysis. The number of blood vessels increased in the skin tissue in contact with the sensor compared to that in the group without the sensor. In contrast, the group in which wireless power transmission and the ECG sensor were operated showed increased vasculature compared to the group in which the sensor was not operated; in addition, visual inspection revealed that the thickness and length of the blood vessels were considerably increased after the operation of the sensor (Figure 2A). In addition, the sensor in the group with wireless-powered transmission showed attachment of the peritoneal muscle to the subcutaneous tissue. These findings confirmed that many blood vessels were formed in the subcutaneous tissues surrounding the sensor and that the portion in contact with the sensor showed increased blood vessel formation compared to the outer portion (Figure 2B). Thus, our results showed that the activation of the wireless-powered transmission system and sensor promoted inflammation in the subcutaneous tissue and was not biocompatible.

### 3.2. Wireless-Powered Transmission Induced Inflammatory Cell Infiltration

There was no inflammatory reaction in the epithelium of the skin into which the sensor was inserted. The subcutaneous tissues of the group in which the sensor was not operated showed that the subcutaneous tissue exhibited partial blood vessel dilation and presence of inflammatory cells. In contrast, in the group receiving wireless-powered transmission, numerous inflammatory cells were observed in the blood vessels and peripheral parts of blood vessels (Figure 2C). To assess the inflammatory response, we examined cell infiltrates in the tissue surrounding the sensor and observed that the total cell number increased by 6.3-fold in the functional sensor group compared to that in the nonfunctional sensor group (Figure 2D). In the tissue surrounding the sensor, many cells were observed in the area contacting the sensor, and a large number of blood vessels were distributed in the periphery (Figure 2E).

### 3.3. Operation of the Wireless Power Transmission Sensor Induced Cell Death by Inflammatory Reaction

**Figure 3:**
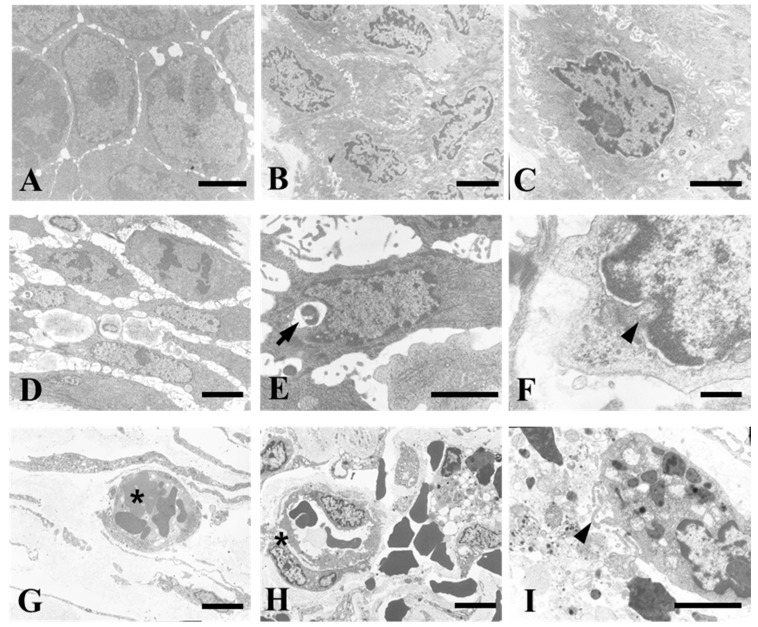
The wireless-powered source induced an inflammatory response and increased the number of immune cells in the surrounding tissue. (**A**) Normal tissues; (**B**,**C**) groups with sensor only; (**D**–**I**) wireless power transmission and sensor operational group (arrow, phagocytosis (**E**); arrowhead, destruction of the nuclear membrane (**F**) and cell membrane (**I**); asterisk, vascular endothelial cells (**H**)). scale bar = 10 µm (**A**–**E**,**G**–**I**) and 2 µm (**F**).

Analysis of the TEM images of the skin and subcutaneous tissue revealed a typical inflammatory response. The skin tissues of the group without sensor insertion were dense with almost no cell gap (Figure 3A). In the absence of sensor activation, the morphology of the cell membrane and nuclear membrane were rough, and more intercellular spaces were observed (Figure 3B,C). The group in which the sensor was functional exhibited signs of apoptosis, including loss of the nuclear envelope and cell membrane, as well as leakage of cell organelles. The sensor attached to the subcutaneous tissue exhibited signs of inflammation and phagocytosis (Figure 3D–I). We demonstrated that the operation of the ECG sensor induced phagocytosis with an inflammatory response in vivo.

### 3.4. Inflammatory Markers were Upregulated Due to the Operation of the Wireless Power Transmission System

**Figure 4:**
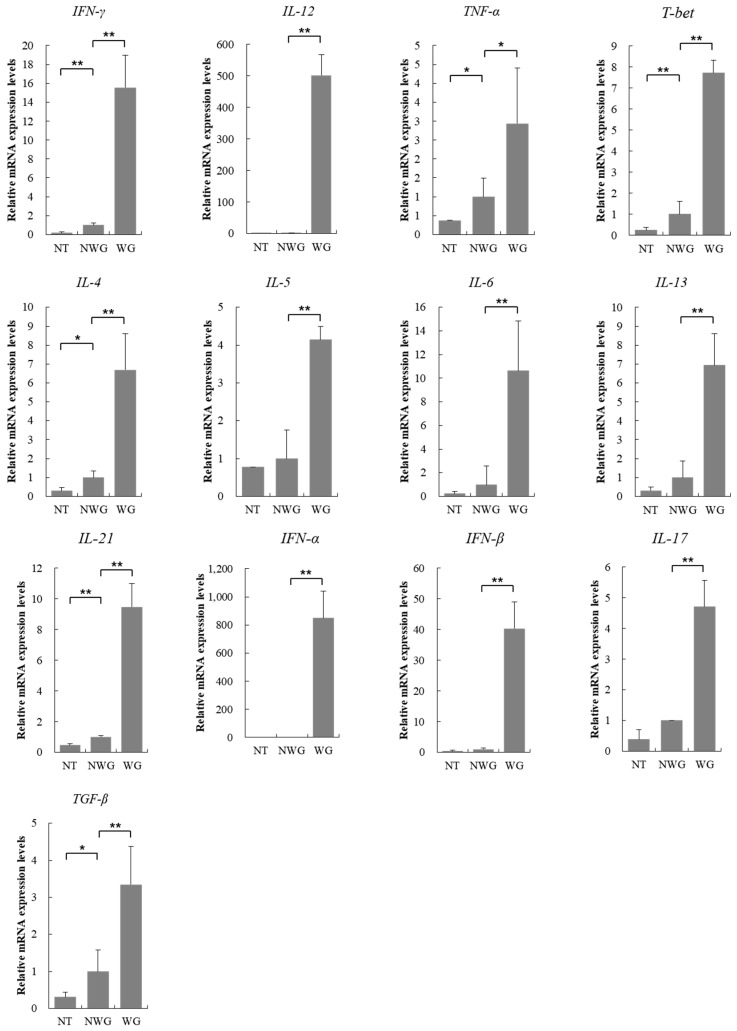
mRNA expression patterns of inflammation markers according to the operation of the wireless-powered transmission system and the sensor (NT, normal; NWG, not working group; WG, working group). Significant difference from NWG, * *p* < 0.05 and ** *p* < 0.01.

For analysis of the inflammatory response, we examined changes in the expression of inflammation-related molecular markers. mRNA analysis revealed that the expression of all inflammatory markers was increased. The mRNA levels of genes encoding the T-helper type 1 (Th1) marker interferon *(IFN)-γ* (15.5-fold), interleukin (*IL)-12* (501.6-fold), tumor necrosis factor *(TNF)-α* (2.9-fold), and *T-bet* (7.7-fold) were increased. In particular, the mRNA levels of *Il12*, which is involved in the differentiation of Th1 cells, was significantly increased compared to that of other markers. The expression of Th2 molecular markers, including *IL-4* (6.7-fold), *IL-5* (4.1-fold), *IL-6* (10.6-fold), and *IL-13* (6.9-fold), were increased. Moreover, the expression of *IL-21* (9.4-fold), *IFN-α* (849.8-fold), *IFN*-*β* (40.3-fold), *IL-17* (4.7-fold), and transforming growth factor *(TGF)-β* (3.3-fold) were also increased (Figure 4). The significant increase in inflammatory response caused by operation of the wireless-powered transfer system and ECG sensor indicated that both the material source and the in vivo operation conditions triggered biocompatibility issues.

## 4. Discussion

The development of wearable devices enables easy and rapid measurement of medical information. Recently, a miniaturized body implantable sensor was utilized for measuring the health information of patients [16]. Moreover, there has been considerable progress in systematization of wireless sensors in various fields, and advancement in technology has dramatically improved the quality of wearable sensors. Thus, interfacing with the most recent wireless device may greatly enhance the efficiency and reduce the sizes of sensors. Despite the substantial progress in the development of implantable in vivo sensors in terms of duration of use and elimination of batteries [9,17], advancements regarding commercialization post insertion of the device is still lacking. In this study, we evaluated the biological safety of wireless-powered sensors in vivo. Specifically, we sought to examine the biocompatibility of the tissue in response to wireless-powered sensor operation. In the intraperitoneal cavity of rats, we compared differences in tissue organization to determine whether the operation of the sensor increased tissue damage compared to groups in which the sensor was not functional. We observed that the operational sensor dramatically increased apoptosis, inflammatory response, cellular and tissue damage, and encapsulation of the tissue surrounding the sensor. These effects were attributed to the wireless power source associated with the operation of the sensor.

Tissue damage due to sensor operation was also reported to induce inflammation and tissue injury, and is assumed to be because of direct contact of tissue with the operating wireless devices [18,19]. In vivo sensors induce greater exposure to electromagnetic waves than ex vivo devices, and wireless power is also known to form an electric field in the tissues surrounding the sensor [20,21,22]. Our results indicated that the electric field of the wireless signal generated by the electronic equipment induced cell death via a number of inflammatory responses. In the subcutaneous tissue, which is comparatively distant from the sensor and the periphery of the sensor, no visible angiogenic or inflammatory reactions were observed. In this experimental model, the part of the device generating wireless power was larger than the sensor part, whereas the part of the device in which the inflammatory reaction was induced was limited to the periphery of the sensor. This induced an inflammatory reaction that was confined to the wireless power transmission system and its proximity to the sensor transmitting and receiving the signals.

Th cells can be classified as Th1 and Th2 types on the basis of the secreted cytokines. Th1 cells, which produce pro-inflammatory cytokines, promote cell-mediated immune responses. Th2 cells, which produce *IL-4*, *IL-5*, *IL-6*, *IL-9*, *IL-10*, and *IL-13*, induce strong antibody response and eosinophil accumulation, secrete Th2-type cytokines, and activate the humoral immune response of B cells. Moreover, these cells also promote IgE production, activate eosinophils, promote B cell growth, and activate inflammatory cells [23]. *IL-12*, produced by activated macrophages or dendritic cells, activates natural killer (NK) cells and is a potent inducer of *INF-γ* production by T cells or NK cells. *INF-γ* activates macrophages, increases the amount of *IL-12*, and promotes Th1-type responses [24]. In addition, *IFN-γ* acts on B cells to stimulate the production of IgG antibodies and assists in the production of cytotoxic T cells. *IL-12* promotes the development of specific immunity by increasing the differentiation of Th1 cells produced by *INF-γ*. Thus, *IL-12* and *INF-γ* play significant roles in protection against intracellular infections [25]. TNF-α acts on macrophages to stimulate inflammatory response and acts as a co-stimulator for activation of T cells and B cells. In this study, we confirmed that the mRNA levels of Th1/2-related cytokines were increased by the operation of the wireless-powered transmission sensor. In particular, the expression of *IL-12* was significantly increased. Thus, we concluded that the wireless-powered transmission system induced a cell-mediated Th1 immune response. Additionally, numerous eosinophils were observed in the peripheral tissue of the sensor, and the levels of Th2-associated cytokines were increased, confirming that the inflammatory response by T cells was induced by wireless-powered transmission. We also observed that the levels of *IL-21*, *IFN-α*, *IFN-β*, *IL-17*, and *TGF-β*, as well as that of T cell-associated inflammatory markers were elevated. This suggested that the operation of the wireless power transmission system or sensor induced an inflammatory response in the body. However, in a study using the same 13.56 MHz radio-frequency power transmission system implant, the numbers of ATP molecules and neutrophils were increased in the early stage. In contrast, the inflammation reaction decreased when the transplanted device was retained for longer durations [26]. These differences may be due to variations in the operating environment of the implants, suggesting that inflammatory responses varied depending on the conditions.

Further studies on the immune system are required to reduce the adverse effects associated with implantation in the human body. Several laboratories have performed biocompatibility studies on the material of implants in the human body and improvements have been achieved. However, studies on the effect of electromagnetic waves generated during the operation of such sensors are limited. In this study, we investigated the in vivo safety of the ECG sensor using a wireless power system, and our observations confirmed that future studies on the safety of implantable medical monitoring systems are required. Implantable sensors are useful for providing care to patients under emergency conditions. Although a number of biocompatible materials have been developed to overcome the immune rejection of grafts, more studies on the effects of wireless power transmission in humans are warranted.

Currently, studies on implant-induced inflammatory response are being conducted continuously. However, most studies have only confirmed the inflammatory response in a fragmented manner, and systematic studies have not been conducted. Although this study has identified related genes, it is not without limitations. First, we did not study whether the cause of the inflammatory response was the wireless ECG sensor or the wireless power source. With the exception of the wireless transmission system, the material, temperature, and operating environment of the sensor have been studied previously. The group without a functioning sensor exhibited weak levels of inflammation approximately 2 months after insertion. However, a large inflammatory reaction occurred after sensor operation for 5 days, without a clear cause. Few of the possible causes include expected transmission and reception of radio waves or the effects of the wireless charging systems. Second, we confirmed that the activity of all inflammatory markers used in the study was increased by the operation of the wireless-powered transmission system. Although inflammatory reactions are triggered by multiple factors, we concluded that the inflammatory reaction observed in the present study was likely based on T cells, predominantly Th1 cells.

Currently, many implant products are in use and many implant materials show excellent performance with little foreign body reaction. This study was conducted to circumvent the health problems associated with the use of battery-operated devices, insertion of which is not permitted in the human body. Various techniques should be developed in future for solving this problem.

## 5. Conclusions

In this study, we investigated the biocompatibility of an ECG sensor using a wireless power transmission system in an animal model. The sensor was encapsulated by the subcutaneous tissue, and we observed induction of angiogenesis, inflammation, and phagocytosis by the wireless power transmission system. Results demonstrated that the induced tissue damage was associated with the operation of wireless power. Further studies on the safety of in vivo wireless power transmission are required.
